# A review on the epidemiology of myopia in school children worldwide

**DOI:** 10.1186/s12886-019-1220-0

**Published:** 2020-01-14

**Authors:** Andrzej Grzybowski, Piotr Kanclerz, Kazuo Tsubota, Carla Lanca, Seang-Mei Saw

**Affiliations:** 1 Department of Ophthalmology, University of Warmia and Mazury, Olsztyn, Poland; 2 Foundation for Ophthalmology Development, Institute for Research in Ophthalmology, Gorczyczewskiego 2/3, 60-554 Poznan, Poland; 3 Private Practice, Gdańsk, Poland; 4 Department of Ophthalmology, Keio University School of Medicine, Tokyo, Japan; 5 Tsubota Laboratory, Inc., Tokyo, Japan; 6 Singapore Eye Research Institute, Singapore, Singapore; 7 Saw Swee Hock School of Public Health, National University of Singapore, Singapore, Singapore; 8 Duke-NUS Medical School, Singapore, Singapore

**Keywords:** Myopia, Epidemiology, Risk factors, Children

## Abstract

**Background:**

Due to high prevalence myopia has gained importance in epidemiological studies. Children with early onset are at particular risk of complications associated with myopia, as progression over time might result in high myopia and myopic macular degeneration. Both genetic and environmental factors play a role in the increasing prevalence of myopia. The aim of this study is to review the current literature on epidemiology and risk factors for myopia in school children (aged 6–19 years) around the world.

**Main body:**

PubMed and Medline were searched for the following keywords: prevalence, incidence, myopia, refractive error, risk factors, children and visual impairment. English language articles published between Jan 2013 and Mar 2019 were included in the study. Studies were critically reviewed for study methodology and robustness of data. Eighty studies were included in this literature review.

Myopia prevalence remains higher in Asia (60%) compared with Europe (40%) using cycloplegic refraction examinations. Studies reporting on non-cycloplegic measurements show exceptionally high myopia prevalence rates in school children in East Asia (73%), and high rates in North America (42%). Low prevalence under 10% was described in African and South American children. In recent studies, risk factors for myopia in schoolchildren included low outdoor time and near work, dim light exposure, the use of LED lamps for homework, low sleeping hours, reading distance less than 25 cm and living in an urban environment.

**Conclusion:**

Low levels of outdoor activity and near work are well-established risk factors for myopia; this review provides evidence on additional environmental risk factors. New epidemiological studies should be carried out on implementation of public health strategies to tackle and avoid myopia. As the myopia prevalence rates in non-cycloplegic studies are overestimated, we recommend considering only cycloplegic measurements.

## Background

The modern rise in myopia mirrors a trend with children in many countries spending considerable amounts of time engaged in reading, studying or — more recently — using computer and smartphones. The evidence suggests that not only genetic, but also environmental factors such as time spent outdoors [1–4], play a major role in this rise, and probably explain the epidemic of myopia that has appeared in East Asia. In other parts of the world, the prevalence of myopia also seems to be increasing. Therefore, myopia has gained particular importance in epidemiological studies. It is estimated that 1.4 billion people were myopic in 2000, and it is predicted that by 2050 the number will reach 4.8 billion [5]. Socioeconomically, refractive errors, particularly if uncorrected, can affect school performance, limit employability and impair quality of life. Myopia is known to be associated with several ocular complications such as retinal detachment, glaucoma, cataract, optic disk changes and maculopathy [6]. High prevalence rates pose a major public health challenge due to visual impairment. The global potential productivity loss associated with the burden of visual impairment in 2015 was estimated at US$244 billion from uncorrected myopia, and US$6 billion from myopic macular degeneration [7]. Children with early onset myopia are the group at major risk, as they will have higher duration of the disease, higher myopia progression and will be at risk of developing high myopia plus myopic macular degeneration. Age of myopia onset or duration of myopia progression is the most significant prognosticator of high myopia in later childhood [8].

The aim of this study is to present a review on the current epidemiology and risk factors for myopia in school children aged 6–19 years.

## Main body

### Methodology

#### Literature search

PubMed and Medline were searched to identify the prevalence of myopia among children, as reported in articles between January 2013 and March 2019. The following keywords were used in various combinations: *prevalence, incidence, myopia, refractive error, and visual impairment* ((“prevalence”[All Fields] OR “incidence”[All Fields]) AND (“refractive error”[All Fields] OR “myopia”[All Fields] OR “visual impairment”[All Fields])). All publications in English and abstracts from non-English publications were reviewed. The reference lists of relevant publications were also considered as a potential source of information. If other studies (e.g., older than 5 years) were essential to draw conclusions, they were included in the discussion section. Studies were critically reviewed for study methodology and robustness of data, particularly the myopia definition and measurements under cycloplegia. No attempts to discover unpublished data were made.

#### Study selection

Full-text articles included in the prevalence analysis were required to meet the following criteria: 1) a cross-sectional or cohort design 2) refractive error measurements taken with a refractometer 3) clear definition of myopia and information on cycloplegic or non-cycloplegic measurements 4) prevalence assessed in children aged 6–19 years 5) studies with a minimum sample of 100 children. If more than one definition of myopia was used in a study, the prevalence for the more commonly used one was selected in order to enable comparison. Results for up to two age-groups were presented, and if data for more than two cohorts were reported, the average for the study or the most common age-group was selected. Studies were excluded from the prevalence analysis if they presented self-reported near-sightedness, reported the prevalence of visual impairment (but not myopia) or included animals.

## Results

The search identified 1627 unique articles. Twenty-eight articles fulfilled the criteria for being included in the main analysis (myopia prevalence). One study was excluded, as it presented data from primary care optometry clinics [9]. Additionally, 55 articles were included in the analysis of risk factors.

### Prevalence of myopia in school children

**Fig. 1:**
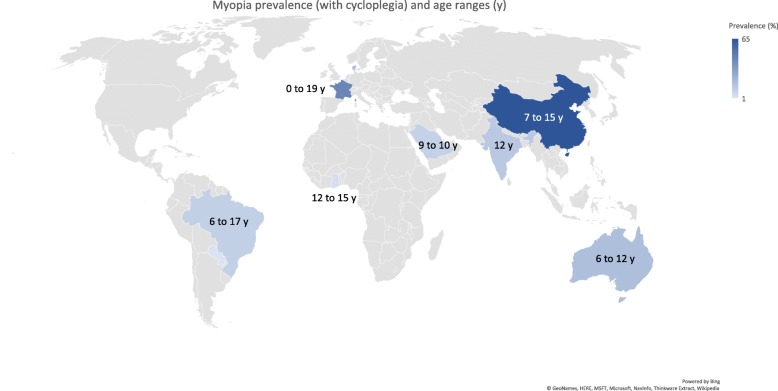
Geographical and age breakdown in myopia prevalence (cycloplegic measurements). Maps were adapted from Bing©GeoNames, HERE, MSFT, Microsoft, NavInfo, Thinkware Extract, Wikipedia

**Fig. 2:**
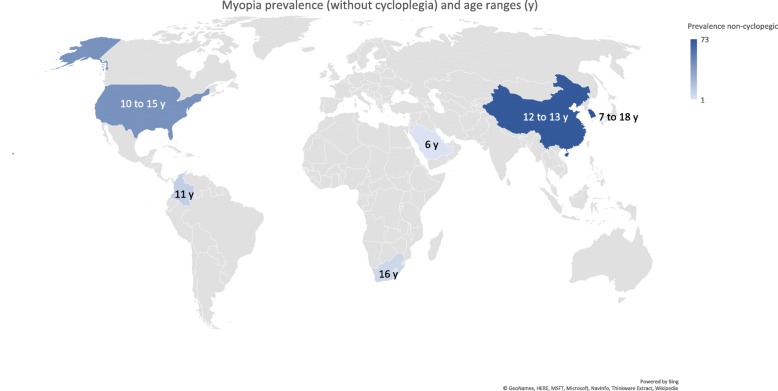
Geographical and age breakdown in myopia prevalence (non-cycloplegic measurements). Maps were adapted from Bing©GeoNames, HERE, MSFT, Microsoft, NavInfo, Thinkware Extract, Wikipedia

The prevalence of myopia was determined by the spherical equivalent refraction (SER) calculated as sphere plus the half of the cylindrical error. The reported prevalence of myopia is shown in Table 1 (list of studies in Additional file 1), with a geographical and age breakdown in Fig. 1 (cycloplegic measurements) and Fig. 2 (non-cycloplegic measurements). The prevalence ranged from 0.7% in Saudi Arabia (children aged 3 to 10 years) [35], 1.4% in South America (children aged 5–15 years) [28] to 65.5% in a cohort of 3rd year junior high school students (age 14–15 years; mean 15.25 ± 0.46 years) in the Haidian district of Beijing. The highest prevalence of myopia in schoolchildren was reported in East Asia and Singapore, urban areas of China, Taiwan and South Korea [39, 40]. In Europe the prevalence rates reached 42.7% in a 10–19-year French cohort [24].

**Table 1:** Cross-sectional studies reporting the prevalence of myopia in school children

Study	Region	Country	Cycloplegia	Definition	Participants	Age range/cohort	Mean Age	Prevalence (95% CI)
Li et al. 2017 [10]	Asia (East)	China	Yes	SE < -0.5 D	37,424	3rd year junior high school	15.25 ± 0.46	65.48% (N/A)
Pan et al. 2017 [11]	Asia (East)	China	Yes	SE < -0.5 D	2346	13–14 years (grade 7)	13.8 ± 0.8	29.5% (27.7–31.4%)
Guo et al. 2016 [12]	Asia (East)	China	Yes	SE ≤ -0.5 D	3055	primary and middle school (grades 1–9)	13.6 ± 1.6	47.4% (45.6–49.2%)
Zhou et al. 2016 [13]	Asia (East)	China	Yes	SE ≤ -0.5 D	3469	follow-up from 6 to 15 years of age	N/A	54.9% (45.2–63.5%)^e^
He et al. 2015 [1]	Asia (East)	China	Yes	SE ≤ -0.5 D	951	6 years	6.6 ± 0.34	39.5% (N/A)
Wu et al. 2013 [14]	Asia (East)	China (East)	Yes	SE ≤ -0.5 D	6026	4–18 years	9.7 ± 3.3	36.9% (36.0–38.0%)
Guo et al. 2015 [15]	Asia (East)	China (West)	Yes	SE ≤ -0.5 D	1565	6–21 years	11.9 ± 3.5	60.0% (N/A)
Lin et al. 2014 [16]	Asia (East)	China (rural)	Yes	SE < -0.5 D	585	6–17 years	10.6 ± 2.5	23.3% (N/A)
Saxena et al. 2015 [17]	Asia (South)	India	Yes	SE ≤ -0.5 D	9884	urban school-children	11.6 + 2.2	13.1% (12.5–13.8%)
Aldebasi et al. 2013 [20]	Asia (Middle East)	Saudi Arabia	Yes	SE ≤ -0.5 D	846	primary school children	9.5 ± 1.8	6.5% (N/A)
Al Wadaani et al. 2013 [21]	Asia (Middle East)	Saudi Arabia	Yes	SE ≤ -0.75 D	2002	6–14 years (primary school children)	9.4 ± 2.3	9.0% (7.7–10.2%)
Lundberg et al. [22]	Europe	Denmark	Yes	SE ≤ -0.5 D	307	average age: 9.7, 11.0, 12.9 and 15.4 years (screened at 1–2,5 year intervals)	15.4 ± 0.7	17.9% (N/A)
Tideman et al. 2017 [23]	Europe	Netherlands	Yes	SE ≤ -0.5 D	5711	6 years	6.37 ± 0.7 (myopia) 6.16 ± 0.5 (non-myopia)	2.4% (N/A)
Matamoros et a. 2015 [24]	Europe	France	Yes	SE ≤ -0.5 D^c^	1781	0–9 years	N/A	19.6% (N/A)
Matamoros et a. 2015 [24]	Europe	France	Yes	SE ≤ -0.5 D^c^	8289	10–19 years	N/A	42.7% (N/A)
Kumah et al. 2013 [25]	Africa	Ghana	Yes	SE ≤ -0.5 D^d^	2435	12–15 years	N/A	3.4% (2.7–4.2%)
Lira et al. 2016 [26]	South America	Brazil	Yes	SE < -0.5 D	778	6–17 years	N/A	9.6% (N/A)
Moraes Ibrahim et al. 2013 [27]	South America	Brazil	Yes	SE ≤ -0.5 D	1590	10–15 years	12.2 ± 1.6 (boys) 12.4 ± 1.6 (girls)	3.14% (2.28–4.0%)
Carter et al. 2013 [28]	South America	Paraguay	Yes	SE ≤ -0.5 D	168	5–16 years	N/A	1.4% (N/A)
French et al. 2013 [29]	Australia	Australia	Yes	SE ≤ -0.5 D	863	6 years (younger cohort)	N/A	14.8% (N/A)
French et al. 2013 [29]	Australia	Australia	Yes	SE ≤ -0.5 D	1196	12 years (older cohort)	N/A	17.3% (N/A)
Guo et al. 2017 [30]	Asia (East)	China	No	SE ≤ -0.5 D	35,745	6–18 years (school-based)	12.6 ± 3.4	70.9% (70.5–71.4%)
You et al. 2014 [31]	Asia (East)	China	No^a^	SE ≤ -0.5 D	15,066	7–18 years (first grade of each school level)	13.2 ± 3.4	64.9% (64.2–65.7%)
Gong et al. 2014 [32]	Asia (East)	China (Beijing)	No	SE ≤ -0.75 D	15,316	7–18 years	12.1 ± 3.3	53.4% (52.6–54.19%)
Rim et al. 2016 [33]	Asia (East)	South Korea	No	SE < -0.5	7486	7–11 years	N/A	48.2% (45.9–50.6%)
Rim et al. 2016 [33]	Asia (East)	South Korea	No	SE < -0.5	7486	12–18 years	N/A	73.0% (71.0–74.8%)
Mahayana et al. 2017 [34]	Asia (South East)	Indonesia	No	SE ≤ -0.5 D	410	N/A (school-based)	10.01 ± 1.84	32.68% (N/A)
Alrahili et al. 2017 [35]	Asia (Middle East)	Saudi Arabia	No	SE ≤ -1.0 D in aged 6 years and more SE ≤ -3.0 D in aged 3–6 years	1893	3–10 years	6.2 ± 1.9	0.7% (N/A)
Hrynchak et al. 2013 [36]	North America	United States of America	No	SE < -0.5D^b^	370	10–15 years	N/A	42.2% (N/A)
Galvis et al. 2017 [37]	South America	Colombia	No	SE ≤ -0.5 D	1228	8–17 years	11.4 ± 2.1	11.2% (9.5–13.0%)
Wajuihian et al. 2017 [38]	Africa	South Africa (rural)	No	SE ≤ -0.5 D	1586	13–18 years (high-school)	15.81 ± 1.56	7% (6–9%)
Total					166,934			

*Abbreviations CI* confidence interval, *N/A* not assessed, *SE* spherical equivalent; ^a^measurement under cycloplegia in 1082 of 15,066 children; ^b^cycloplegic measurements in about 3% of patients; ^c^cycloplegic autorefraction in children, non-cycloplegic refraction in adults, age range not presented; ^d^measurements under cycloplegia if unaided visual acuity was 20/40 or worse; ^e^presented as a population-based cross-sectional survey, however, the results report that the prevalence rate among initial emmetropes and hyperopes, after 5 years

Compared with cycloplegic measurements the majority of the studies reporting on myopia prevalence with non-cycloplegic measurements reported much higher prevalence rates. For example, a prevalence of 73% was found in South Korean children aged 12 to 18 years old [33]. However, there are some countries where the prevalence rates remain low, such as Brazil (3.14 and 9.6%) [26, 27] and Ghana (3.4%) [25]. In these countries even when considering an overestimated non-cycloplegic measurements the prevalence of myopia in school children remains low e.g., in the Republic of South Africa (7%) [38] or in Colombia (11.2%) [25, 37]. Carter et al. also found a very low prevalence of myopia (with relatively common hyperopia) in indigenous schoolchildren from Paraguay (1.4%) [28].

A critical parameter for epidemiological analysis of myopia is age, as prevalence rates of myopia are known to increase significantly with age (Table 1). For example, in the Shandong Children Eye Study, only 1.76 ± 1.2% of four-year-old children had myopia, while at the age of 17 years the prevalence was 84.6 ± 3.2% [14]. In another study, the one-year incidence of myopia among grade 1 (age 6-7 years) Chinese students was 33.6% (95% CI: 31.7–35.%), with a progression rate of -0.97 D (95% CI: -1.22 to -0.71 D) [41]. Moreover, myopia beginning at school continues to progress up to adulthood in almost half of the patients [42].

### Change over time

In several countries the prevalence of myopia has increased in the last years. In a study from the Haidian District in Beijing, China, the prevalence of myopia in a cohort of 15-year-old schoolchildren increased from 55.95% in 2005 to 65.48% in 2015 [10]. In Fenghua city, eastern China, the prevalence of myopia in high school students increased from 79.5% in 2001 to 87.7% in 2015, and high myopia (SER greater than − 6.0 D) was a major contributor to this increase [43]. In Western China not only myopia prevalence increased, but also a higher rate of annual myopia progression was recently noted [13]. The Waterloo Eye Study showed a long-term increase in myopia prevalence also in the United States [36]. The prevalence rate reached 42.4% in 10 to 15-year-old children, and 53.9% in 15 to 20-year-old; this was significantly higher than the 21% peak value (in those aged 20–30 years) reported in a comparable study done in 1892 [43].

### Risk factors

Risk factors influencing the prevalence of myopia are presented in Table 2. There are some risks factors that can contribute for the prevalence increase such as parental myopia, ethnic differences, less time outdoors, increased near work, population density and socioeconomic status.

**Table 2:** Risk factors for the prevalence of myopia in the analyzed studies

Risk factor	Country where the study was conducted
Female gender	China [10, 12, 14, 15] Colombia [37] India [17] Saudi Arabia [21, 35]
Low outdoor activity	Australia [27] China [1, 15] Netherlands [23]
Parental myopia	Australia (6-year-old cohort) [29] China [12, 32, 45] Finland [42] India [17] Japan [46]
Increasing age	Brazil [26] China [12, 14, 15] India [17] Poland [18, 19] Saudi Arabia [21, 35]
Time spent on near work/studying	Australia [29] China [12, 32] India (over 5 h daily) [17] Taiwan [47]
Higher socio-economic status	India [17]
Low family income	Netherlands [23]
Higher body mass index	Japan [46] Netherlands [23]
Use of LED lamps for homework (compared to incandescent or fluorescent lamps)	China [11]
Urban environment, high population density and small home size	China [14] Hong Kong [48] Indonesia [34]
Rural environment	Saudi Arabia (only in girls) [21]
Private schooling and watching TV over 2 h daily and playing mobile/video games	India [17]
Low sleeping hours	China [32]
Lower vitamin D levels, less participation in sports and foreign descent	Netherlands [23]
Westernized dietary habits	Japan [46]

### Parental myopia

In a study conducted by Lim et al. children (aged 6–18 years) with two myopic parents had a mean refractive error of − 2.33 D and the odds ratio of having myopia in childhood with two myopic parents was 2.83, compared with no parental myopia [45]. Although genetic factors have some impact on eye growth, the development of myopia appears to be mainly influenced by environmental factors such as education [49]. Data from the Handan Offspring Myopia Study the children’s refraction was similar to that of their parents at the age of 14 [16]. The inter-generational myopic shift was estimated to be only 1 D at 18 years of age. Thus, it might be concluded that environmental factors such as education influence emmetropization [49]. Looking at between-sibling refractive error in 700 families from the United States, Jones-Jordan et al. found that environmental factors reduced the estimated refractive error correlation between siblings by only 0.5% [50]. This was confirmed by the Collaborative Longitudinal Evaluation of Ethnicity and Refractive Error Study [51]. Less hyperopic and more myopic refractive error at the ages of 7 to 13 years was consistently associated with myopia onset, while having myopic parents, near work and time outdoors were not. The SAVES study revealed that parental myopia was a risk factor for myopia only for the 6-year-old children, but not in 12-year-old cohort [29].

A recent study from Netherlands found seven independently parameters associated with faster axial elongation (AL) in children with 6 to 9 years of age: parental myopia, 1 or more books read per week, time spent reading, no participation in sports, non-European ethnicity, less time spent outdoors, and baseline AL-to-Corneal Radius ratio [52]. Based on the aforementioned results, the authors suggested that behavioural changes are of the highest importance in these children, and employing preventive measures should be considered.

### Outdoor time

Outdoor time has been proven to be the strongest environmental factor that can delay myopia onset. The Sydney Adolescent Vascular and Eye Study (SAVES) evaluated the risk factors for the incidence of myopia in Australian schoolchildren during a 5–6 year follow-up period in two cohorts: younger (*n* = 892; aged 6 years at baseline) and older (*n* = 1211; aged 12 years at baseline) [29]. The children that became myopic spent less time outdoors compared to those who remained nonmyopic (16.3 vs 21.0 h in the younger cohort, *p* < 0.0001; and 17.2 vs 19.6 h in the older cohort, *p* = 0.001). The Avon Longitudinal Study of Parents and Children confirmed the negative association between the time outdoors and myopia; additional the time outdoors in 3 to 9 years-old age range was associated with reduced incidence of myopia at the age of 10 to 15 years [53]. Another study showed that patterns of daily outdoor light exposure differed substantially between Australian (105 ± 42 min/d) and Singaporean children (61 ± 40 min/d; *p* = 0.005) [54].

Myopia progression was not strongly associated either with near work or outdoor/sports activity in siblings with common environmental exposures [50]. In a randomized clinical trial by He et al., a 40-min class of outdoor activities on each school day for 3 years resulted in a reduced incidence of myopia from 39.5 to 30.4% [1]. A recent RCT showed that outdoor activities can inhibit progression in myopic children aged 6 to 7 years old by 30% in 1 year [2]. These result might indicate that high-risk patients require a sum of treatments for the control of the condition, including changes in lifestyle (increase in outdoor time) and treatment with atropine eye drops, progressive contact lenses or orthokeratology.

Interesting results were presented in a recent study on 16–19-year-old Norwegian Caucasians (*n* = 393) living in 60° latitude North, where autumn-winter is 50 days longer than summer [55]. In their investigation the total time spent doing outdoor was not associated with myopia (3.65 ± 1.5 h in myopes, and 3.81 ± 1.9 in non-myopes, *p* = 0.64). Moreover, the prevalence of myopia was quite low (13% for SER lower than ≤ − 0.5 D), despite the few daylight hours in the autumn-winter period (10 h 36 min–11 h 5 min) and high levels of indoor activity and near work [55]. The commonly agreed underlying mechanism of time spent outdoors proposed by researchers is based on the release of retinal dopamine that controls scleral growing and remodeling. Genetic observations add credence to the current notion that myopia is caused by a retina-to-sclera signaling cascade that induces scleral remodeling in response to light stimuli [56]. However, it is possible that other variables may influence emmetropization, including ultraviolet light [57] or blue-light [58]. Moreover, a recent systematic review found that lower blood vitamin D concentrations are associated with increased risk of myopia; on the other hand serum vitamin D levels may be just a proxy for time outdoors [59]. Viewing distances are also much greater outdoors, with the accommodative requirements being smaller and giving a more uniform dioptric space [60]. Animal studies provided evidence that sustained hyperopic defocus, which is generated indoors, promotes local eye growth and myopia [61].

The Childhood Health, Activity, and Motor Performance Eye Study determined the association between physical activity and myopia; in a group of 307 Danish children accelerometer measurements were conducted at mean ages of 9.7, 11.0, 12.9 and 15.4 years [22]. The prevalence of myopia at the final time-point was 17.9% and was not associated with physical activity. In an American cohort, exercise was associated with a lower rate of myopia [62]. Tideman et al. found that myopic children (*n* = 5711, six-year-old children) in Europe spent less time outdoors, have lower vitamin D3 and higher body mass index than non-myopic children [23]. Similarly, Terasaki et al. analysed lifestyle factors related to myopia progression in the third-year elementary school students in Japan [46]. High body weight, parental myopia and Westernized dietary habits were associated with increased myopia prevalence. In Finland higher myopia during maturity was related to parental myopia, less time spent on sports and outdoor activities during school-years [42]. However, less time spent on sports might also be a proxy of low outdoor activity. Another recent investigation reported a relationship between myopia and BMI, with obese children having higher risk of developing myopia [63]. These results should be interpreted carefully as obese children may engage less in exercise and outdoor activities, as this may be a confound factor that needs further research. Those represent an important potentially modifiable risk factor that may be a target for future public health efforts, involving the protection of children not only from myopia, but also from other unhealthy behaviors that can impact health.

Translation of research findings, regarding outdoor time, to clinical practice is also growing rapidly. A recent questionnaire applied to paediatric ophthalmologists all over the world show that 86 % of the respondents advised children with myopia to spend more time outdoors [64].

### Near work

The SAVES study revealed that near work was a risk factor for myopia but only for the 6-year-old children, and not in 12-year-old cohort [29]. This result might indicate that near work can be a factor for inducing the earlier onset of myopia in smaller children. There might be a difference in the mechanism of setting myopia development between early onset and later onset myopes. Children who became myopic performed significantly more near work (19.4 vs 17.6 h), which was statistically significant (*p* = 0.02), however, the association was lower than for outdoor activity. A combination of both factors may be implicated in the myopia development. Shorter reading distance and higher myopia at the baseline exam (1-year prior to the final assessment) were risk factors for myopia progression in a cohort of second-grade primary school (age 7–8 years) children in Taipei [47]. In this study, fast myopia progression in children was associated with more myopia at baseline and shorter reading distance. Similarly, in a study of primary and middle school-aged pupils in Guangzhou (*n* = 3055, mean age of 13.6 ± 1.6 years), children whose reading distance was less than 25 cm were more likely to have myopia than those reading from a distance of 25–29 cm or over 29 cm (*p* < 0.001) [12]. In the same study, reading for more than 2 hours daily was positively associated with myopia in boys, while spending time watching television per week was associated with myopia in girls. Taiwanese children attending private classes outside the regular school system in the evening or on the weekends for ≥2 h/d had increased risk of myopia occurence [65]. The authors hypothesize that this effect may be due to increase near visual activity or reduced time outdoors. Because the effect of near distance activities on myopia onset and progression was shown to be higher in younger children, it seems to be reasonable to limit unnecessary time spent on near distance activities (including electronic devices) by pre-school children, and these activities should be under strict parental control.

### LED lamps and hours of sleep

In a study by Pan et al., conducted on 2346 Chinese children aged 13 to 14-years-old using LED lamps for homework after school had a higher prevalence of myopia (SER less than − 0.75 D) and longer axial length than those using incandescent (*p* = 0.04 and *p* = 0.007, respectively) or fluorescent lamps (*p* = 0.02 and *p* = 0.003, respectively) [11]. Gong et al. found low hours of sleep to be an independent risk factor for myopia in 15,316 students of mean age of 12.1 ± 3.3 years from 18 districts of Beijing. Children with 7 hours or less of sleep (odds ratio 3.37, 95% confidence interval (CI): 3.07–3.70, *p* < 0.001) or about 8 h of sleep (odds ratio 2.12, 95% CI 1.94–2.31, p < 0.001) had a higher risk compared to those who slept 9 hours or more daily [32]. A disadvantage of the study was that it analysed non-cycloplegic SER. The mechanism underlying the sleeping time-myopia relationship is not well understood yet and future research is needed; presumably inactivity of the ciliary muscle during the sleep could prevent or alleviate the myopic progression. However, other new variables may be involved such as the effect of dim light. New findings suggest that in addition to bright light exposure, rod pathways stimulated by dim light exposure could be important to human myopia development [66]. One study with Australian children aged 10 to 15 years old demonstrated that myopic children spent less time in both scotopic and outdoor photopic light conditions compared with non-myopic children. Myopes may also have reduced sensitivity to low spatial frequency S-cone stimuli with consequences in their failure to emmetropize normally [67].

### Population density

Higher population density seems to be associated with myopia risk, independent of time spent outdoors and other environmental factors [68]. High population density and small home size was also associated with longer axial length and refractive error in children in Hong Kong and Beijing [48, 69]. The Beijing study reported other risks factors that are associated with myopia, such as urban region of habitation [69]. In a cohort of 12-year-old children from the urban region of greater Beijing the prevalence of myopia was 70.9% [30]. The mean refractive error in 18-year-olds was − 3.74 ± 2.56D. The prevalence of myopia was highest in provincial capitals in Hubei province, followed by non-provincial cities, and the lowest in rural areas, with a statistically significant difference (*p* < 0.05) [70]. On the other hand, the incidence of myopia in a rural area in southwestern Japan was very low, from 0.3 to 4.9% over a five-year study in the late 1990s [71]. In general, high population density might be a surrogate of outdoor and near work; children in urban areas might spend less time outdoors, as they might may not have available places where to play.

### Socioeconomic status (SES)

In the North India Myopia Study, the prevalence of myopia was 13.1% [17]. Myopia was more common among children with higher SES and among private school students, compared to governmental school pupils. Presumably children in private schools spend more hours at school compared to children in public schools; they spend more time reading and writing at home, with significantly more pressure and a greater likelihood of extra classes. Studying and reading for over 5 hours daily, watching television for over 2 hours daily, and playing video/mobile games were also significantly associated with myopia. In this study there was no obvious mechanism linking higher SES and attending private schools to myopia, except through the education that the children received. A plausible hypothesis would be that children from higher SES families and private schools would be getting more intensive education, as within the study children from private schools spent more time reading at home than those from government schools (*p* < 0.001).

Contradictory findings were reported by a Dutch study of a multi-ethnic cohort of 6-year-old children, revealing a significant influence of socioeconomic factors on the prevalence of myopia [23]. In particular, children of non-European descent, with children from low maternal education, low family income, were more likely to be myopic. These findings are in contrast to the results cited earlier in North Indian children [17]. However, children from families with a non-European ethnic background, similarly to those in private schools in North India, spend lower time outdoors [23]. The children in the Rotterdam study are still very young, and the effects of education are unlikely to be clear, and although their parents have low incomes, they might have a greater commitment to education as a pathway to success.

## Discussion

Cycloplegic refraction is established as the gold standard for epidemiological studies on refractive errors. Nevertheless, within our review nine studies used non-cycloplegic measures, while 19 studies presented cycloplegic refraction. Studies reporting non-cycloplegic measurements of prevalence cannot be considered as reliable; application of non-cycloplegic measurements leads to substantial errors, both in prevalence rates and associations with risk factors [77, 72]. For example, Lundberg et al. reported the prevalence of myopia in children reaching 33.6% using non-cycloplegic measurements and 17.9% under cycloplegia [22]. In the Shandong Children Eye Study the difference between cycloplegic and non-cycloplegic SER was 0.78 ± 0.79 D; this difference decreased with age and increased with greater hyperopic refractive error [73]. In the study by Fotedar the difference between cycloplegic and non-cycloplegic SER was 1.18 D (95% CI: 1.05–1.30 D) for 6 year-old-children, and 0.84 D (0.81–0.87 D) for 12-year-old-children [74]. Thus, the refractive error was misclassified in 9.5% 6-year-old children, and 17.8% 12-year-old children [74]. Interestingly, the Beijing Myopia Progression Study (which enrolled children aged 6 to 17 years) found that a major difference between non-cycloplegic and cycloplegic was associated with the progression of myopia in children, but not with the onset of myopia [75].

The different studies included in this review used different definitions of myopia. Most studies define myopia as a SER less than or equal to − 0.5 D. Some studies use a criterion of SER less than − 0.5 D, or less than or equal to − 0.75 D. Myopia was also defined as a SER less than or equal to − 1.0 D in children aged 6 years. A SER greater than or equal to − 3.0 D in children aged 3–6 years was reported in one study [35]. The definition of myopia is of extreme importance, and even small changes in the threshold definition (±0.25D) have been shown to affect significantly the conclusions of epidemiological studies [76, 77, 78]. Recently, the International Myopia Institute suggested employing a ≤ − 0.5 D threshold as an evidence based consensus [79].

Another issue is the choice of eye; usually measurements of the right eye are included in the analysis. In one study conducted in Seoul, South Korea, the age standardized prevalence was reported to be has high as 80% in children aged 12–18 years [33]. However, myopia was classified according to the Korea Centers for Disease Control and Prevention; it employs definition of myopia as a SER greater than or equal to − 0.75 D in either (worse) eye. When applying a definition of <− 0.5 D in the right eye, the prevalence rates dropped down to 73%. Moreover, one should consider that these results were significantly biased as the measurements were done without cycloplegia.

Interestingly, in some migrant groups, primarily of East Asian origin, the children were significantly more myopic than those of European origin, presumably because of the intensive education that the children are receiving [80, 81]. Children of East Asian ethnicity spend less time outdoors and more time in near work activities compared to European Caucasian children at all school ages [80]. Rudnicka et al. found that the increase in myopia prevalence over the last decade is related with ethnic differences, with only a small change seen in whites but a significant increase observed in East Asians and a weaker increase among South Asians [82]. Myopia was also common in a diverse Southern Californian pediatric cohort and children of Asian ancestry had the highest prevalence. Particular lifestyle habits in different populations may partially explain dissimilarities in myopia prevalence [62]. It has been suggested that a probable causative role in the development of myopia is the competitive and stressful education systems in some East Asian countries [83].

New risk factors, apart from outdoor time, such as the use of LED lamps for homework, dim light, low sleeping hours, reading distance less than 25 cm and living in an urban environment were described in recent studies. Additional epidemiological studies should be carried out to further expand the knowledge of outdoors on myopia progression. Interventional studies might be also needed to better understand the effectiveness of preventive methods in different settings and age groups. Although light intensity patterns in humans have been implicated in myopia protection, research needs to be further expanded to understand how bright needs to be the exposure to avoid myopia. Longitudinal patterns of light exposure in different refractive errors (e.g. myopes, hyperopes and emmetropes) are needed to understand which of light parameters is the most important (e.g. light intensity, duration or regularity). This study did not focus on the prevalence of high myopia, which is an important indicator and should be further developed.

## Conclusion

It can be concluded that prevalence rates were shown to increase in Asia, but also in Europe and North America. Particular lifestyle habits in different populations may partially explain dissimilarities in myopia prevalence between geographical regions. Preventive measures such as outdoor programs and changes on near distance activities in preschool children should be implemented.

## Supplementary information

**Additional file 1.** The list of cross-sectional studies reporting the prevalence of myopia in school children.

## Data Availability

N/A (review article).
