# Supplementary material for: A review on the epidemiology of myopia in school children worldwide
Source: BMC Ophthalmol. 2020 Jan 14;20:27. doi: 10.1186/s12886-019-1220-0 (PMC6961361; doi:10.1186/s12886-019-1220-0)
Supplement: Supplementary file 1 — Additional file 1. The list of cross-sectional studies reporting the prevalence of myopia in school children. [file 12886_2019_1220_MOESM1_ESM.pdf]

Appendix 1. The list of cross-sectional studies reporting the prevalence of myopia in school children

Cycloplegic:

- Li Y, Liu J, Qi P. The increasing prevalence of myopia in junior high school students in the Haidian District of Beijing, China: a 10-year population-based survey. BMC Ophthalmol 2017;17:88.
- Pan C-W, Wu R-K, Liu H, et al. Types of Lamp for Homework and Myopia among Chinese School-Aged Children. Ophthalmic Epidemiol 2017:1–7.
- Guo L, Yang J, Mai J, et al. Prevalence and associated factors of myopia among primary and middle school-aged students: a school-based study in Guangzhou. Eye 2016;30:796–804.
- Zhou W-J, Zhang Y-Y, Li H, et al. Five-Year Progression of Refractive Errors and Incidence of Myopia in School-Aged Children in Western China. J Epidemiol 2016;26:386–395.
- He M, Xiang F, Zeng Y, et al. Effect of Time Spent Outdoors at School on the Development of Myopia Among Children in China. JAMA 2015;314:1142.
- Wu JF, Bi HS, Wang SM, et al. Refractive error, visual acuity and causes of vision loss in children in Shandong, China. The Shandong Children Eye Study. PLoS One 2013;8:e82763.
- Guo K, Yang DY, Wang Y, et al. Prevalence of myopia in schoolchildren in Ejina: the Gobi Desert Children Eye Study. Invest Ophthalmol Vis Sci 2015;56:1769–1774.
- Lin Z, Gao TY, Vasudevan B, et al. Generational difference of refractive error and risk factors in the Handan Offspring Myopia Study. Invest Ophthalmol Vis Sci 2014;55:5711–5717.
- Saxena R, Vashist P, Tandon R, et al. Prevalence of myopia and its risk factors in urban school children in Delhi: the North India Myopia Study (NIM Study). PLoS One 2015;10:e0117349.
- Aldebasi YH. Prevalence of correctable visual impairment in primary school children in Qassim Province, Saudi Arabia. J Optom 2014;7:168–176.
- Al Wadaani FA, Amin TT, Ali A, et al. Prevalence and pattern of refractive errors among primary school children in Al Hassa , Saudi Arabia. Glob J Health Sci 2013;5:125–134.
- Lundberg K, Suhr Thykjaer A, Søgaard Hansen R, et al. Physical activity and myopia in Danish children-The CHAMPS Eye Study. Acta Ophthalmol 2017.
- Tideman JWL, Polling JR, Hofman A, et al. Environmental factors explain socioeconomic prevalence differences in myopia in 6-year-old children. Br J Ophthalmol 2017:bjophthalmol – 2017–310292.
- Matamoros E, Ingrand P, Pelen F, et al. Prevalence of Myopia in France: A Cross-Sectional Analysis. Medicine 2015;94:e1976.

- Kumah BD, Ebri A, Abdul-Kabir M, et al. Refractive error and visual impairment in private school children in Ghana. Optom Vis Sci 2013;90:1456–1461.
- Lira RPC, Arieta CEL, Passos THM, et al. Distribution of Ocular Component Measures and Refraction in Brazilian School Children. Ophthalmic Epidemiol 2017;24:29–35.
- Moraes Ibrahim F, Moraes Ibrahim M, Pomepo de Camargo JR, et al. Visual impairment and myopia in Brazilian children: a population-based study. Optom Vis Sci 2013;90:223–227.
- Carter MJ, Lansingh VC, Schacht G, et al. Visual acuity and refraction by age for children of three different ethnic groups in Paraguay. Arq Bras Oftalmol 2013;76:94–97.
- French AN, Morgan IG, Mitchell P, et al. Risk factors for incident myopia in Australian schoolchildren: the Sydney adolescent vascular and eye study. Ophthalmology 2013;120:2100–2108.

Non-cycloplegic

- Guo Y, Duan JL, Liu LJ, et al. High myopia in Greater Beijing School Children in 2016. PLoS One 2017;12:e0187396.
- You QS, Wu LJ, Duan JL, et al. Prevalence of myopia in school children in greater Beijing: the Beijing Childhood Eye Study. Acta Ophthalmol 2014;92:e398–e406.
- Gong Y, Zhang X, Tian D, et al. Parental myopia, near work, hours of sleep and myopia in Chinese children. Health 2014;06:64–70.
- Rim TH, Kim S-H, Lim KH, et al. Refractive Errors in Koreans: The Korea National Health and Nutrition Examination Survey 2008-2012. Korean J Ophthalmol 2016;30:214–224.
- Mahayana IT, Indrawati SG, Pawiroranu S. The prevalence of uncorrected refractive error in urban, suburban, exurban and rural primary school children in Indonesian population. Int J Ophthalmol 2017;10:1771–1776.
- Alrahili NHR, Jadidy ES, Alahmadi BSH, et al. Prevalence of uncorrected refractive errors among children aged 3-10 years in western Saudi Arabia. Saudi Med J 2017;38:804–810.
- Hrynchak PK, Mittelstaedt A, Machan CM, et al. Increase in myopia prevalence in clinic-based populations across a century. Optom Vis Sci 2013;90:1331–1341.
- Galvis V, Tello A, Otero J, et al. Refractive errors in children and adolescents in Bucaramanga (Colombia). Arq Bras Oftalmol 2017;80:359–363.
- Wajuihian SO, Hansraj R. Refractive Error in a Sample of Black High School Children in South Africa. Optom Vis Sci 2017;94:1145–1152.
